# A Novel Multilayer Natural Coating for Fed-State Gastric Protection

**DOI:** 10.3390/pharmaceutics14020283

**Published:** 2022-01-26

**Authors:** Rober Habashy, Mouhamad Khoder, Abdullah Isreb, Mohamed A. Alhnan

**Affiliations:** 1 School of Pharmacy and Biomedical Sciences, University of Central Lancashire, Preston PR1 2HE, UK; robo_85@hotmail.com (R.H.); AIsreb@uclan.ac.uk (A.I.); 2 Drug Discovery, Delivery and Patient Care (DDDPC) Theme, School of Life Sciences, Pharmacy and Chemistry, Kingston University London, Kingston Upon Thames, London KT1 2EE, UK; 3 Centre for Pharmaceutical Medicine Research, Institute of Pharmaceutical Science, King’s College London, London SE1 9NH, UK

**Keywords:** delayed release, gastric protection, probiotics, GRAS, hypochlorhydria

## Abstract

Several nutraceutical products require gastric protection against the hostile environment in the stomach. Currently marketed synthetic and semi-synthetic coatings suffer from major shortcomings such as poor gastric protection, slow-release response to pH change, and the use of artificial ingredients. The challenge of coating natural products is further exacerbated by the relatively high gastric pH in the fed state. In this work, a novel natural enteric coating is presented as a breakthrough alternative to current solutions. Two coating systems were devised: (i) a triple-layer coating that comprises a wax layer embedded between two alginate-based coatings, and (ii) a double-layer coating, where an overcoat of organic acids (fumaric or citric acid) is applied to an alginate-based coating. The multi-layer architecture did not impact the pH-responsive nature of the coating even when more biologically relevant Krebs bicarbonate buffer of lower buffer capacity was used. Interestingly, the gastric protection barrier of organic acid-based coating remained resistant at elevated gastric pH 2, 3, or 4 for 2 h. This is the first report of using an alginate-based coating to provide gastric protection against fed-state stomach conditions (pH 2–4). Being biodegradable, naturally occurring, and with no limit on daily intake, the reported novel coating provides a superior platform to current coating solutions for pharmaceutical and nutraceutical products.

## 1. Introduction

The nutraceuticals market has significantly grown in the past decade and $278.96 billion was a benchmark estimate globally of 2021. Such rapid growth was attributed to the increased demands for functional healthy dietary intake and functional supplements [1]. Several nutraceutical products need gastric protection, e.g., fibres, oils, minerals, vitamins, or probiotics. To meet this market demand, different enteric coatings were developed as commercially available solutions to provide nutraceutical coatings using GRAS grade semi-synthetic (e.g., Nutrateric^®^ and Aquateric^®^) and synthetic (e.g., Eudraguard^®^ Biotic and Control) polymers.

Crafting enteric coating from naturally occurring ingredients offers several advantages such as avoiding restriction on daily intake, biodegradability [2], and consistency with many nutraceutical products that claim no artificial additives. However, achieving such challenging enteric coating criteria using naturally occurring materials proved to be an elusive task. Shellac has long been presented as a viable natural coating solution [3]. However, it suffers from major batch-to-batch variations and a slowed drug release upon storage [2]. Several products such as Protect^TM^ and Swanlac^®^ were marketed based on ammoniated shellac with the addition of alginate to regulate shellac dissolution in the intestinal phase [4]. However, such strategies proved to have limited success due to a slow dissolution particularly at pH 6.8 [2].

Several reports indicated the pH of the stomach to be at 1-3 in the fasted state and to exceed pH 3 in the fed state [5]. As many nutraceutical products are consumed with food, the challenge of enteric coating is exacerbated by the need to provide gastric protection at the higher pHs in the fed-state gastric environment. In addition, patients receiving multiple doses of proton pump inhibitors (PPIs) might have a gastric pH of >4 [6]. A medium of pH 4 has been proposed to be used to simulate the fed state in the gastric environment [7,8].

One significant implication of elevated gastric pH is the fast ionisation of the hydrophilic weak acid, e.g., alginic acid (pKa 3.4) [9], leading to a premature disintegration of the enteric coating in the gastric environment [10]. In this context, shellac-based coating provided gastric protection at elevated gastric pH. However, shellac-based systems showed slow responses at pH 7.4 in both phosphate and bicarbonate buffers [11]. In addition, alginate-based coatings such as Nutrateric^®^ and Aquateric^®^ failed to provide sufficient protection at elevated pHs (fed state) [4,12]. More recently, methacrylate-based coating to provide enteric production was introduced by Evonik under Eudraguard Control^®^ and Biotic^®^. Nevertheless, their synthetic nature might significantly limit their use in the nutraceutical market that is often conscious of using only natural ingredients.

Several examples in the literature indicated the use of multi-layer designs for enteric coatings to enhance their performance. For instance, double coating has been used to achieve sustained release for tablets [13] and pellets [14]. Adding neutralised acidic polymeric layer underneath the enteric coating was used to accelerate the pH response of the enteric coating in small intestine medium upon gastric emptying [15,16]. This strategy was also applied to safeguard bacteria- and pH-sensitive coating response to colonic environment [17]. 

Waxes are conventionally used at the end of the coating process of tablets to provide a glossy finish. Previous attempts to employ waxes via hotmelt coating to provide enteric-coated pellets achieved limited success. While coating with stearic and palmitic acids provided a relatively limited release at pH 1.2, the coated pellets released the drug at pH 2–4 and demonstrated a slow pH response following pH change [18]. However, the use of wax in multiple coating layers is yet to be explored.

We have previously reported the use of fatty glycerides dispersion in aqueous alginate solution to provide a pH-dependent release profile [10]. More recently, an O/W emulsion of waxes in alginate solution was developed to boost the gastric-resistant properties of the coated films [4]. The reported coating showed a significant pH-responsive enteric coating in comparison to commercially available synthetic and semi-synthetic coating solutions. However, there is still the need to improve the performance of this coating film against fed-state gastric conditions. In this work, we have adopted a multilayer coating architecture using alternating layers of alginate, wax, or organic acids to achieve highly pH-responsive delayed drug release. Here, we propose the addition of waxes or an organic acid layer to our previously reported alginate-based coating as a novel formulation approach to improve the gastric protective properties of the coating, particularly under elevated gastric pH condition. In this strategy, pure wax layers were hypothesised to act as a hydrophobic barrier to decrease moisture intake, while organic acids such as citric acid are used to regulate the local pH of the dissolved enteric coating.

## 2. Materials and Methods

### 2.1. Materials

Anhydrous theophylline was purchased from Acors Organics. The excipients used for tablet compression were: directly compressible lactose monohydrate—Lactopress (BASF SE, Ludwigshafen, Germany), polyvinylpyrrolidone K90 (Sigma-Aldrich, Dorset, UK), microcrystalline cellulose (MCC) PH101 (FMC Biopolymer, Woluwe-Saint-Lambert, Belgium), cross-carmellose sodium SD-711 (FMC Biopolymer, Belgium), and magnesium stearate (Sigma–Aldrich Co., Ltd., Dorset, UK). The coating components were: sodium alginate (Alg, 15–20 cps) supplied by Sigma-Aldrich (Dorset, UK), ceresin wax supplied by Fisher Scientific (UK), glyceryl monostearate (Imwitor^®^ 900K, GMS) donated by Cremer OLEO (Hamburg, Germany), citric acid monohydrate and pectin from citrus supplied by Sigma-Aldrich (USA), and fumaric acid purchased from Fischer Scientific (USA).

### 2.2. Model Tablet Preparation

Theophylline tablets were selected as a model core for coating as a relatively bulky tablet (~600 mg) to mimic typical commercially available nutraceuticals and food supplement tablets. The tablets were prepared by wet granulation method as described earlier [10]. The tablets were compressed using a Riva Minipress single-punch tablet press (Riva, Argentina, South America) at a crushing strength of a nominal value of 120 N.

### 2.3. Preparation of Coating System

#### 2.3.1. Casted Film

Casted films were prepared by blending a weight ratio of sodium alginate, ceresin wax, and GMS of 10:6:1 and dispersing them at a concentration of 2.25% *w*/*v* in 70 °C water under magnetic stirring at 150 rpm for 30 min. The obtained emulsion (20 mL) was poured into Teflon-coated 10 cm circular trays and dried in a fan oven at 40 °C for 24 h. The obtained films were peeled off the trays, cut in 1 × 1 cm square and stored in sealed polybags for further tests.

#### 2.3.2. Coated Tablets

Multilayer coatings, either bilayer or triple-layer, were obtained using fluidised bed coater and/or drum pan coater. The main coating layer was alginate-based (Alg), while pure wax and organic acid-based (fumaric acid (Fum) or citric acid (Citr)) layers were applied as a sub-coat (SC), an overcoat (OC), or a middle layer. Table 1 describes the formulations of different coating layers.

While F1 is a ceresin wax-based monolayer coating obtained by drum coater, F2 is a single coating made of O/W wax-Alg emulsion, using GMS as emulsifier and a Strea-1 fluidised bed coating (GEA Pharma Systems AG, Aeromatic-Fielder, Bubendorf, Switzerland) to achieve a 10% coating weight gain as previously detailed [4]. Both F3 and F4 are bilayer coatings, and F1 coating was added either as a sub-coat (SC) or an overcoat (OC) to F2 Alg-based coating, respectively. F5 coating is a triple-layered coating consisting of three layers: two F2 coatings (SC and OC) and an F1 coating as a middle layer. Finally, F6 and F7 are bilayer coatings where an organic acid-based layer (fumaric acid (F6) or citric acid (F7)) was applied as OC of F2. 

Wax coating (F1) was obtained using drum pan coater (Erweka, Germany). Tablets were warmed for 2 min using a hot-air generator to achieve a target temperature of ~65 °C. The temperature was continuously monitored using an infrared sensor. Molten wax at 80 °C was then poured into tablets to achieve a 5% WG. The drum speed was set at 250 rpm for 5 min to allow wax film to adhere to the model core. Coated tablets were then transferred to a fluidised bed coater (GEA Pharma Systems AG, Bubendorf, Switzerland) and wax layer was cured at a temperature of 45 °C for 10 min to achieve smooth and homogenous distribution of the applied wax coating on the tablet surface.

For organic acid-based coating layers (F6 and F7), the organic acids were dissolved at 5% *w*/*v* in a pectin solution (1% *w*/*v*) and applied using Strea-1 fluidised bed coater to achieve a weight gain of 5% of tablet weight. The inlet air temperature was adjusted at 55 °C and outlet air temperature was 45 °C, yielding a tablet bed temperature of 42–45 °C. The atomizing pressure was about 350 mBar. The coating solution was stirred at 70 °C and the spray rate was about ~3.4 mL/min.

### 2.4. Model Tablet Preparation

Thermogravimetric analysis (TGA) was carried out using TA Analysis Q500 analyser (TA Instruments, Hertfordshire, UK) where a ThermoScan was set from 25 to 500 °C [19]. Accurately weighed samples (10 mg of Alg, GMS, ceresin wax, physical mixture, fumaric or citric acid) were placed in 40 μL aluminium pans and scanned at a heating rate of 10 °C/min. The experiments were carried out under a nitrogen gas flow of 40 and 60 mL/min for furnace and sample, respectively. The thermal degradation profile was analysed using TA Universal Analysis 2000 software (TA Instruments, Hertfordshire, UK). 

Differential scanning calorimetry (DSC) analysis was carried out using TA Analysis 2000 (TA Instruments, Hertfordshire, UK). Samples (7 mg of individual ingredients, their physical mixture, and wax-containing casted films) were accurately weighed in T0 pan and analysed from 25 °C to 150 °C (most natural waxes’ melting points lie between 40–140 °C) at a heating rate of 10 °C/min. To cover the organic acid melting point, organic acid DSC analysis was run up to 350 °C. Samples were preheated to 100 °C for 5 min to exclude the effect of humidity then cooled to −10 °C. Analysis was carried out under a purge of nitrogen (50 mL/min). The data were analysed using TA 2000 analysis software (TA Instruments, Hertfordshire, UK). All measurements were carried out in triplicate.

### 2.5. Morphology of the Coating

Cross-sectioned coated tablets were examined using a Quanta-200 scanning electron microscope (SEM) at 20 kV. Samples were placed on metallic stubs and gold-scattered under vacuum for 2 min using JFC-1200 Fine Coater (Jeol, Tokyo, Japan), prior to imaging.

### 2.6. Disintegration Test

The disintegration test was conducted in accordance with United Stated Pharmacopeia 31 standards [20]. Tablets (6 units) were placed in a ZT122 disintegration tester (Erweka, Germany) operated for one hour in 0.1 M HCl, then the medium was replaced with pH 6.8 phosphate buffer, as specified in USP 31 [20]. 

### 2.7. Water Uptake

In order to evaluate the gastric resistance and protection capabilities of the coating system, six tablets were weighed and then placed for 1h in 800 mL 0.1 M HCl at 37 °C. The tablets were drained of excess acid using filter paper. The wet weight was recorded, and water uptake was calculated according to Equation (1):

(1) $$\mathrm{water}\;\mathrm{uptake}\;\%=\left(\frac{\mathrm{wet}\;\mathrm{mass}-\mathrm{dry}\;\mathrm{mass}}{\mathrm{dry}\;\mathrm{mass}}\right)\times 100$$

### 2.8. In Vitro Drug Testing

#### 2.8.1. pH Change Release Studies Method

The in vitro dissolution of the theophylline from coated tablets was assessed using an AT7 Smart dissolution USP II apparatus (Sotax, Aesch, Switzerland). The dissolution medium was stirred at a paddle rotation of 50 rpm at 37 ± 0.5 °C. The tablets were firstly tested in 750 mL of acidic phase (0.1 M HCl, pH 1.2) for 2 h, followed by 4 h intestinal phase of pH 6.8 (by adding 250 mL of 0.21 mM of tribasic sodium phosphate solution). The percentage of released theophylline was determined at 5 min intervals using UV/vis spectrophotometer (PG Instruments Ltd., Leicestershire, UK) at the wavelength of 272 nm and path length of 1 mm. Data were analysed using IDISis software version 3.0 (Automated Lab, Berkshire, UK).

#### 2.8.2. Krebs’s Bicarbonate Buffer Test

Theophylline release was tested in Krebs’s bicarbonate buffer following the same method detailed above. Following acidic phase (0.1 M HCl, pH 1.2) for 2 h, the tablets were extracted and placed for 4 h in 1000 mL bicarbonate physiological buffer [1]. 

#### 2.8.3. Release at Elevated Gastric pH

Theophylline release from coated tablets was tested in elevated gastric pH values using the same dissolution system as detailed above, where phosphate buffers pH 2, 3, and 4 were prepared as detailed in British Pharmacopoeia (BP) and used instead of acidic phase (0.1 M HCl, pH 1.2). 

### 2.9. Statistical Analysis

Statistical significance was determined using the one-way analysis of variance (ANOVA) or Student’s *t*-test as appropriate. All experiments were performed in triplicate, and values were expressed as the mean ± standard deviation. Values of *p* < 0.05 were considered statistically significant.

## 3. Results and Discussion

Wax-based coatings were previously explored for their gastro-resistant properties; however, this resulted in a slow response to pH change [18]. In this work, wax coating was applied at the core at 5% WG (F1). In addition to failing to construct the gastro-resistant barrier, F1 delayed and/or slowed drugs’ release in intestinal medium (Supplementary Figure S1). In our recently published study, alginate emulsion-based coatings (corresponding to F2 formulation in Table 1) were able to provide excellent gastro-resistant properties (at pH 1.2) and instant release upon pH increase in SIF [4]. Similarly, alginate and cellulose-derivative-based coatings were able to resist gastric pH 1.2 [2]. These coatings showed limited gastric protective properties at pH values 2–4.

**Figure 1:**
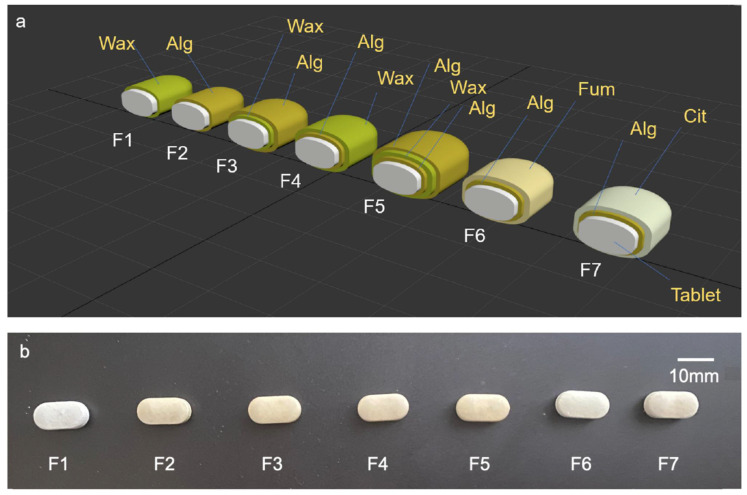
(**a**) Schematic diagram and (**b**) photographs of different natural coating architects.

In this work, we aimed enhance the gastric properties of alginate-based coatings at higher gastric pH values by devising a multilayer coating structure using wax or organic acids as SC or OC in combination with alginate-based coating. Table 1 summarises the compositions of the different coatings investigated in this study. Figure 1a provides a schematic diagram and the photographs of different natural coating architects (Figure 1b).

### 3.1. Morphology and Characteristics of Coated Tablets

**Figure 2:**
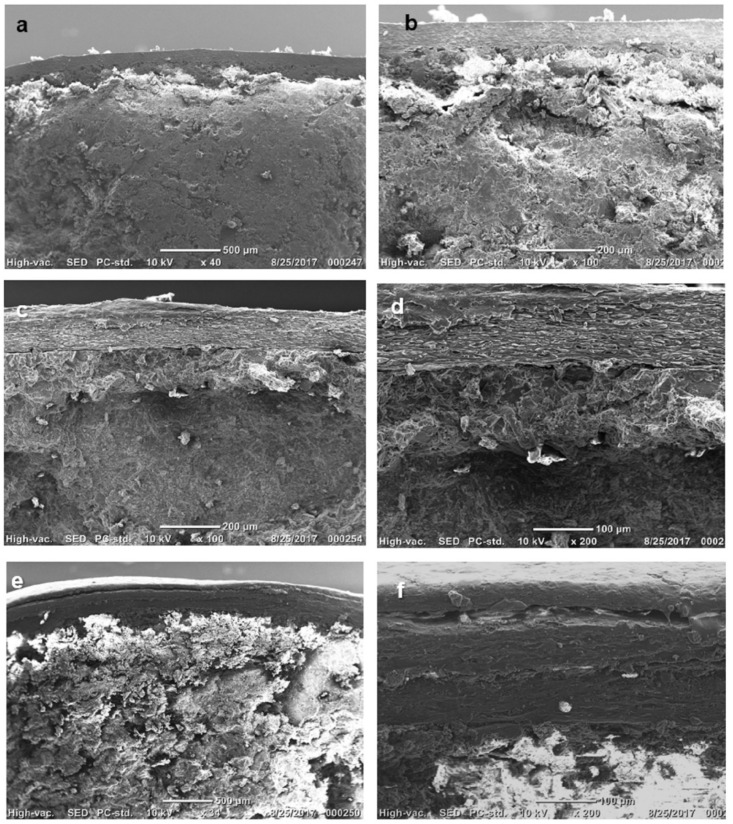
SEM images of cross-sections of Alg-based coating (F2) at (**a**) ×40 and (**b**) ×100 magnifications, Alg based coating overcoated with wax (F3) at (**c**) ×100 and (**d**) ×200 magnifications, (**e**) triple layers of Alg-Wax-Alg (F5) at (**e**) ×34 and (**f**) ×200 magnifications.

Tables coated with F2–F5 showed a yellow tint coating that was evenly distributed over the tablet surface, while F1, F6, and F7 showed a white matte finish. The colour of tablets coated with F6 was white matte (Figure 1). The SEM images of the cross-section of coated tablets illustrated a single layer coating in F2 (Figure 2a,b) of approximately 80–100 µm, two distinctive layers of approximately 80 µm thickness in F3 coating (Figure 2c,d), and three layers of 50, 80, and 100 µm for inner, middle, and outer layer, respectively, in F5 (Figure 2e,f). 

**Figure 3:**
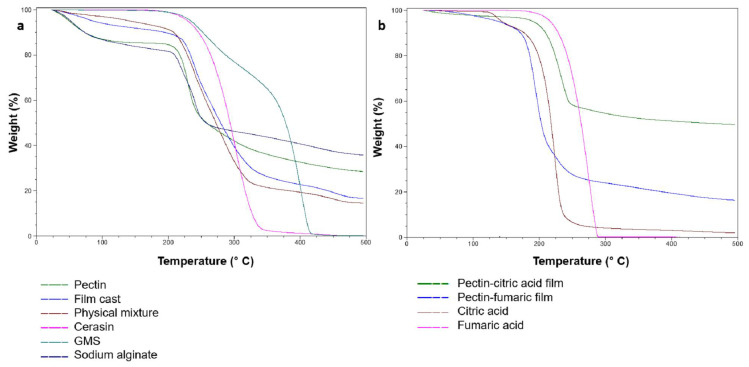
TGA thermographs of (**a**) single components of coating system, physical mixture, and casted film (F2); (**b**) organic acids and their casted film.

**Figure 4:**
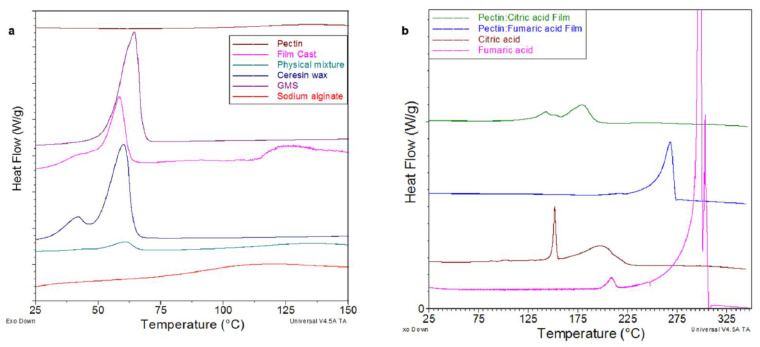
DSC thermographs of (**a**) single components of coating system, physical mixture, and casted film (F2); (**b**) organic acids and their casted film.

The TGA thermographs of raw materials and casted films are shown in Figure 3a,b. Casted films of similar compositing to coating layers were used to allow thermal analysis investigation. No signs of thermal degradation of raw materials and casted films were observed at preparation temperatures (up to 75 °C), suggesting their stability during the coating process. All samples exhibited significant weight losses between 200 and 300 °C that could be attributed to thermal degradation at these higher temperatures. Alginate and pectin weight loss observed at approximately 100 °C could be attributed to the evaporation of moisture content [21]. The DSC thermographs of raw materials and casted films are shown in Figure 4a,b. DSC thermographs of ceresin wax and GMS showed endothermal peaks at approximately 59 and 64 °C, corresponding to their melting points, respectively [22,23]. The DSC thermograms of casted films showed a distinctive endothermic peak at approximately 58 °C, which corresponds to the melting point of ceresin wax [4]. The DSC thermograph for alginate and pectin (Figure 4a) confirmed their amorphous nature with the absence of a melting point [24]. Furthermore, a weight loss in the region of 100 °C was observed, suggesting the evaporation of moisture contents [25]. Fumaric (Fum) and citric (Citr) acid-casted films showed endothermic peaks at 270 and 140 °C, respectively. These peaks matched the melting points of both acids, indicating that a significant portion of the organic acids is in a crystalline form within the pectin structure.

### 3.2. Acid Uptake and Disintegration

The gastric resistance and swelling properties of studied coating systems are listed in Table 2. Tablets coated with F1 did not display sufficient gastro-resistant properties and disintegrated in <20 min in the gastric medium. Composed of water-insoluble ester and fatty acid [26], waxes can act as a hydrophobic barrier and contribute to the resistance to disintegration in an aqueous environment [27].

F2 coating yielded <5% acid uptake and resisted gastric acidity (pH 1.2) for 2 h, with <5% drug release. F2 coating is based on alginate emulsion, whose carboxylate groups remain unionised in acidic gastric fluid. This renders alginate coating insoluble in the acidic gastric medium (pH 1.2), allowing the establishment of a gastro-resistant barrier [28]. F3 showed a 1 h resistance in the gastric acid medium (pH 1.2) before coating rupture. In fact, F3 coating consists of two layers: a wax SC and an alginate emulsion OC. However, the wax SC poorly adhered to the tablet surface, and the structure ruptured in the acidic medium. On the other hand, the Alg-based coating in F4 was directly applied on the model core surface that achieved a better adhesion before being overcoated with a wax layer. However, the wax layer (OC) in F4 broke after one hour in acid phase. Increasing the thickness of the wax layer was explored to enhance its mechanical properties; however, this yielded a slow-release profile (data not shown). Therefore, applying an Alg-based overcoat to F4 (i.e., F5) was envisaged to provide a more resistant film without compromising its pH-responsive properties and to prevent the separation of wax layer observed in F3 and F4. The gastric resistance of F5 was observed up to 2 h with an acid uptake percentage of <5%.

Another alternative approach was developed through the application of a pH regulating outer layer consisting of organic acid coating (F6 and F7). This approach yielded double-layered coatings utilising pectin film comprising organic acids (fumaric or citric acid) as an OC on top of F2 coating. Sodium alginate was not selected for film formation since it was insoluble in the presence of fumaric or citric acid. Pectin was selected as an alternative due to its aqueous solubility and excellent film-forming properties [29]. The incorporation of organic acids (fumaric acid in F6 and citric acid in F7) did not seem to compromise the gastric resistance properties of the coatings at pH 1.2 with an acid uptake of 6 ± 1.9% and 2.9 ± 0.48%, respectively. Furthermore, the drop in water uptake of F7 was significantly lower than what was achieved with the corresponding single-layer formulation (F2) (*p* < 0.05).

### 3.3. In Vitro Drug Release Study

The dissolution profiles of the coated tablets were tested in phosphate buffer as well as in the more biologically relevant bicarbonate buffer (Krebs) [30,31].

- Gastric pH 1.2

**Figure 5:**
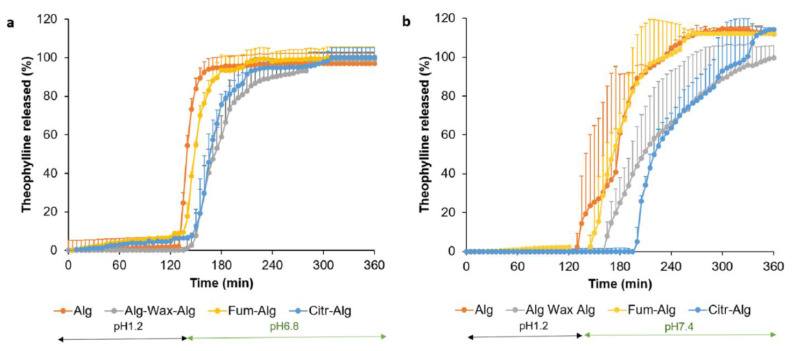
In vitro theophylline release from tablets coated with (Alg (F2), Alg-Wax-Alg (F5), Fum-Alg (F6,) and Citr-Alg (F7)) in acid stage followed by (**a**) intestinal phosphate buffer pH 6.8; (**b**) Krebs’s bicarbonate physiological buffer pH 7.4.

In line with its acid uptake and disintegration behaviour, the wax-based formation (F1) prematurely released ~96% of the drug content within the first 30 min in the gastric medium (pH 1.2) (Supplementary Figure S1). Similarly, since both F3 and F4 failed the disintegration test as the coating dislodged in 1 h, they were also excluded from the release studies. On the other hand, all remaining formulations (F2 and F5–F7) resisted gastric medium (pH 1.2) with <5% of drug release in for 2 h (Figure 5a,b).

b. Intestinal release

Upon changing the release medium pH using phosphate buffer, F2 allowed an immediate release with >80% theophylline released in <45 min. F5, consisting of three layers, showed a delayed and slower release in phosphate buffer with <80% release within 80 min of pH change (Figure 5a). This corresponds to the significantly longer disintegration time of the triple coating in comparison to Alg-based single coating (F2) (*p* < 0.05).

Overcoating F2 with pectin/organic acids layer, using fumaric acid in F6 and citric acid in F7, resulted in a gastric resistance of 2 h, similar to that of F2. However, upon introducing the phosphate buffer (pH 6.8), F7 displayed a slightly slower release compared to F2 and F6 with >80 release within 70 min in the case of F7 or 45 min for F2 and F6 (Figure 5a). Fumaric acid is an acid that is poorly soluble in water (~0.6% *w*/*v*) with a pKa of 3.03 [32]. On the other hand, citric acid is a highly water-soluble tri-carboxylic acid (59.2 g/100 mL) [33] with three pKa values of 3.1, 4.8, and 6.4 [34]. The aim of incorporating organic acids (with low pKa values) into the OC was to prevent excessive alginate ionisation at elevated gastric pH values (see Section 3.3 c). Owing to its higher solubility, the fast and significant dissolution of citric acid in phosphate buffer of pH 6.8 seemed to lower the local pH of alginate SC, resulting in less ionisation of alginate carboxylate groups, hence the slower overall release in the intestinal medium. 

Krebs’s buffer provides a more biologically relevant dissolution medium that better simulates the buffer ionic strength and composition of the small intestine fluid. Following the gastric phase, a lag time of theophylline release of up to 65 min was observed in Krebs’s buffer. This could be attributed to Krebs’s lower buffer capacity that disfavours the ionisation of carboxylic groups of alginate SC in the intestinal medium [31,35], hence the delay in the drug release. A higher variability was observed in the release of the coated tablets when tested in bicarbonate-based buffers compared to that in compendial phosphate buffer. This could be attributed to the slower dissolution of the acidic polymer in mediums with lower buffer capacity (i.e., Krebs). It is possible that the impact of minor variability on film thickness or the presence of weak points within the film structure may be magnified with the slower dissolution of the enteric coating in the bicarbonate buffer. In contrast, the enteric film dissolves at a relatively fast rate in phosphate buffer, such that subtle differences between batch units could be masked. The superior ability of bicarbonate buffers to discriminate different enteric coatings compared to compendial phosphate buffers has previously been reported [31].

It is worth mentioning that F6 and F7 displayed longer lag times of theophylline release, which might indicate the fumaric and citric acids dissolution in Krebs’s buffer that acidify the SC microenvironment and further delayed alginate coating ionisation. Interestingly, F7, comprising citric acid in the OC, showed the longest lag time (up to 65 min), which could be attributed to the high aqueous solubility of citric acid.

c. Fed state gastric pH

Stomach pH increases considerably after a meal (i.e., fed state) [36]. Since most enteric coatings rely on pH-responsive polymers, elevated stomach pH values are more challenging to overcome as enteric coating polymers tend to undergo significant ionisation in the stomach, leading to premature release. 

**Figure 6:**
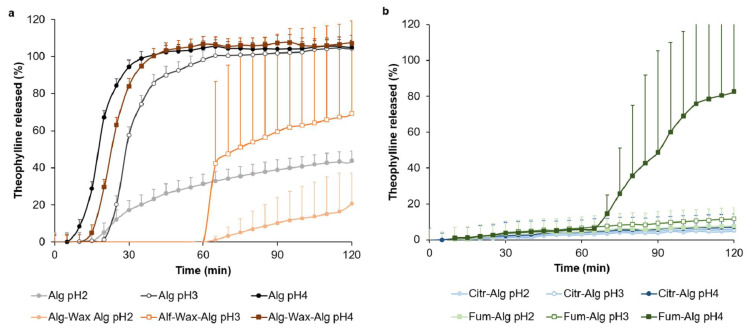
In vitro theophylline release from tablets coated with (**a**) single-layer Alg (F2) and triple-layer Alg-Wax-Alg (F5); (**b**) Fum-Alg (F6) and Citr-Alg (F7) in elevated gastric pH 2, 3, and 4.

Formulations F2 and F5–F7 were assessed for release at elevated gastric pH. F2, consisting of alginate coating, failed to show gastric resistance functionality at elevated gastric pH with ascending release as pH increases with >80% of theophylline released in <30 min at pH 4 (Figure 6a). Alginate is composed of (1–4)-β-d-mannuronic acid (1–4)-α-l-guluronic acid units with a pKa value of 3.45 [9]. The polymer will go through a substantial ionisation at fed state (pH 2–4) leading to fast water imbibition and dissolution [10]. Therefore, a modification in the structure is needed to prevent water penetration. To achieve this, the triple layers coating (F5) was employed to show an improvement of gastric resistance (up to 1 h) at pH 2 and 3 but rapidly released theophylline at pH 4. The marginal improvement of F5 gastric resistance gained at pH 2 and 3 could be attributed to the presence of the hydrophobic wax middle layer and the increased overall diffusion path that ultimately delayed the gastric medium penetration and drug diffusion across the coating. 

Incorporating organic acid in the OC of F6 and F7 yielded a significant improvement of the overall gastro resistance coating capacity at elevated pH values. F6 resisted pH 2 and 3 for 2 h with <10% drug release in gastric medium (Figure 6b) and >80% released within 45 min upon pH change (data not shown). However, at pH 4, F6 underwent a premature release after 1 h with >80% of drug released in gastric medium. Interestingly, using citric acid (F7) resulted in an excellent gastric resistance even at pH 4 (<10% release after 2 h) (Figure 6b). Moreover, >80% of theophylline was released in <45 upon pH change (data not shown).

The application of organic acids containing OCs to resist the fed state’s elevated gastric pH values is a novel approach. The ionisation/dissolution of these organic acids within the OC pectin layer is expected to form a lowered pH local environment that prevents the excessive ionisation of alginate in the SC layer, hence delaying the drug release. While citric acid-containing OC (F7) yielded an excellent gastric barrier across all investigated elevated pHs, Fum acid-containing OC (F6) failed to resist the highest elevated gastric pH (i.e., pH 4). In fact, the better solubility and higher pKa values of citric acid help ionise it more efficiently across the different elevated gastric pH values, leading to a significant lowering of the local pH of the SC alginate layer. However, being poorly soluble in water with lower pKa, fumaric acid underwent limited ionisation in pH 4 that subsequently led to a marginal local acidifying of the alginate coating. Consequently, a significant alginate ionisation might happen in pH 4, leading to a premature drug release in gastric medium. 

The ability of F6 and F7 to resist elevated gastric pH using low cost commercially available ingredients is unique among natural coating systems. Previous reports indicated that Nutrateric^®^ showed limited or no ability to resist higher pH [2]. Likewise, other alginate-based systems, such as Aquateric^®^, and shellac-based coatings, such as Protect^™^ and Swanlac^®^, provided a premature or slow-release response to pH change [4]. Despite the advantages of the reported approach, this needs to be balanced with the additional cost and production complexity of applying multiple coating layers in comparison to a simpler single-layer system. However, the coating process utilises low-cost, commercially available ingredients.

The presented method provides a significant advance in natural coating solutions. As many nutraceutical products are consumed with food, an enteric coating that is able to withstand the relatively elevated pH in the fed state is highly desirable. The citric and fumaric acids that were utilised in this work are naturally occurring and widely used in food and nutraceutical products. Owing to the simplicity of the manufacturing approach that does not involve any organic solvents, as well as the minimal equipment requirements, the coating technique can be widely adopted and used for the production of enterically coated nutraceuticals without artificial ingredients.

## 4. Conclusions

In this study, a novel multilayer coating based on naturally occurring polymers was reported. A wax layer embedded between two Alg-based coatings acted as a hydrophobic barrier and enhanced the overall gastric-resistant acidic medium (pH 1.2). Overcoating with pectin-containing organic acids (fumaric or citric acids) showed superior gastric protection in the fed state (pH 2, 3, and 4) while maintaining a prompt response to pH change. The latter system provides a valuable alternative to current commercially available GRAS, semisynthetic and synthetic enteric coating systems, which fail to provide sufficient protection at the gastric pH in the fed state and provide a rapid response to pH change following gastric emptying. While applying multi-layer coatings may implicate operational complexity and incur additional costs, the evident enhancement to the value of the finished product should not be underestimated.

## Figures and Tables

**Table 1:** Summary of the composition of single layer wax (F1), alginate-based (F2), and multi-layer (F3–F7) coatings.

Coating	Wax (F1)	Alg (F2)	Alg-wax (F3)	Wax-Alg (F4)	Alg-wax-Alg (F5)	Fum-Alg (F6)	Citr-Alg (F7)
**Description**	5% WG wax	10% WG Alg	Wax 5% WG as SC + Alg 10% as OC	Alg 10% WG as SC + Wax 5% WG as OC	Alg 7% WG as SC + Wax 5% WG as mid-layer + Alg 7% WG as OC	Alg 10% WG as SC + 5% fumaric acid as OC	Alg 10% WG as SC + 5% citric acid as OC

WG: weight gain; SC: sub-coat; OC: overcoat.

**Table 2:** A summary of water uptake, acid resistance, and release characteristics of single-layer wax (F1), alginate-based (F2), and multi-layer (F3–F7) coatings.

Material Name	Wax (F1)	Alg (F2)	Alg-wax (F3)	Wax-Alg (F4)	Alg-wax-Alg (F5)	Fum-Alg (F6)	Citr-Alg (F7)
**Gastric Phase**							
Acid uptake (%)	Opened	5 ± 1.49%	4.6 ± 1.2%	4.9 ± 1.6%	3.5 ± 0.78%	6 ± 1.9%	2.9 ± 0.48%
Acid medium resistance	Opened after 20 min	Resisted 2 h	Opened after 10 min	Resisted 1h then ruptured	Resisted 2 h	Resisted 2 h	Resisted 2 h
Drug release in acid medium (120 min)	100%	4.75%	100%	4.9%	0%	6.8%	4.88%
Lag time in intestinal stage (min)	Opened	-	-	Opened	25	15	15
**Intestinal Phase**							
80% release time in buffer stage (min)	-	30	-	-	85	35	70
Disintegration test	Open after 20 min	Resisted 2 h	Resisted 1 h then ruptured	Resisted 1 h then dislodge of the coat	Resisted 2 h	Resisted 2 h	Resisted 2 h
Disintegration time of all tablets in SIF (min)	NA	10.2 ± 1.1	NA (opened in acid phase)	11.3 ± 0.84	22.5 ± 3.0	9.8 ± 0.89	10.4 ± 0.68

## Supplementary Materials

The following supporting information can be downloaded at: https://www.mdpi.com/article/10.3390/pharmaceutics14020283/s1, Figure S1: In vitro dissolution studies of natural coating system in acid stage pH1.2, Wax (F1), Alg-Wax (F3).
