# Supplementary material for: A Novel Multilayer Natural Coating for Fed-State Gastric Protection
Source: Pharmaceutics. 2022 Jan 26;14(2):283. doi: 10.3390/pharmaceutics14020283 (PMC8879697; doi:10.3390/pharmaceutics14020283)
Supplement: Supplementary file 1 [file pharmaceutics-14-00283-s001.zip › pharmaceutics-1503678-supplementary.pdf]

pharmaceutics

# Supplementary Materials: A Novel Multilayer Natural Coating for Fed-State Gastric Protection

**Rober Habashy, Mouhamad Khoder, Abdullah Isreb and Mohamed A. Alhnan**

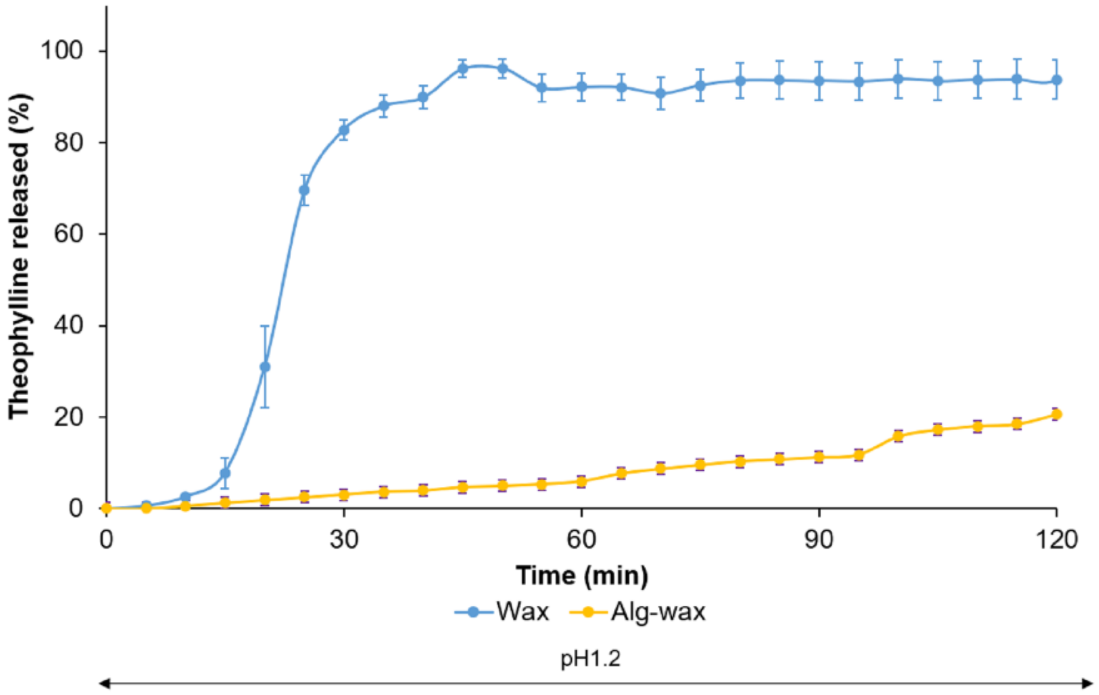


**Figure S1.** In vitro dissolution studies of natural coating system in acid stage pH1.2, Wax (F1), Alg-Wax (F3).
